# Development and Validation of GC-ECD Method for the Determination of Metamitron in Soil

**DOI:** 10.1155/2015/592763

**Published:** 2015-02-05

**Authors:** Shishir Tandon, Satyendra Kumar, N. K. Sand

**Affiliations:** Department of Chemistry (Division of Agricultural Chemicals), College of Basic Sciences and Humanities, G. B. Pant University of Agriculture and Technology, Pantnagar 263145, India

## Abstract

This paper aims at developing and validating a convenient, rapid, and sensitive method for estimation of metamitron from soil samples.Determination andquantification was carried out by Gas Chromatography on microcapillary column with an Electron Capture Detector source. The compound was extracted from soil using methanol and cleanup by C-18 SPE. After optimization, the method was validated by evaluating the analytical curves, linearity, limits of detection, and quantification, precision (repeatability and intermediate precision), and accuracy (recovery). Recovery values ranged from 89 to 93.5% within 0.05- 2.0 *µ*g L^−1^ with average RSD 1.80%. The precision (repeatability) ranged from 1.7034 to 1.9144% and intermediate precision from 1.5685 to 2.1323%. Retention time was 6.3 minutes, and minimum detectable and quantifiable limits were 0.02 ng mL^−1^ and 0.05 ng g^−1^, respectively. Good linearity (*R* ^2^ = 0.998) of the calibration curves was obtained over the range from 0.05 to 2.0 *µ*g L^−1^. Results indicated that the developed method is rapid and easy to perform, making it applicable for analysis in large pesticide monitoring programmes.

## 1. Introduction

Injudicious usage of pesticides may lead to accumulation of residues in the soil. Certainly, the residue levels should not be allowed to exceed the maximum limits. Increasing awareness of the potential impact of such toxic chemistries has led to the development of new and safer methods for its determination to ensure minimum risk to man and environment [1, 2].

Metamitron [4-amino-3-methyl-6-phenyl-1,2,4-triazine-5(4H)-one] belonging to triazinone group was introduced by Bayer AG under the code number BAY DRW1139 with trade mark “Goltix” and protected by USP3847914. It is selective systemic herbicide used as pre- and/or postemergence in sugar beet and beet crops for controlling turf grass, wide range of grasses, broadleaves weeds, and other meadows such as rye. It is applied at 2.0–5.0 kg a.i. ha^−1^ for controlling weeds. It inhibits the photosynthesis by interrupting photosystem-II in susceptible weeds. Half-life of metamitron in soil is less than 5 to more than 14 weeks depending on soil temperature and moisture conditions [3]. According to WHO, metamitron belongs to class III pesticide having slightly hazardous acute toxicity.

Little information is available in the literature regarding metamitron analysis by analytical methods in soil and food commodities. Determination of metamitron by various analytical methods, namely, bioassay method [4], polarography [5], voltammetric [6, 7], vibrational spectrometry [8], HPLC [9–15], GC [16], and LC-MS/MS [17], has been reported.

As most of the laboratories do not have/use the facility of gas/liquid chromatography tandem mass spectrometry for routine analysis and there is very scarce literature on analysis of metamitron in soil by GC, therefore, a sensitive analytical gas chromatographic method capable of estimating in microquantities is required. The objective of this study is to improve the extraction procedure and develop simple, sensitive method which can be conveniently used for the detection and determination method of metamitron and provide information to laboratories that analyse metamitron contamination in environment or are interested in it. The analysis is carried out for the first time in different agroclimatic soil and condition, that is, the subtropical condition of Terai region soil of Uttarakhand, India.

## 2. Material and Methods

### 2.1. Instruments

Hewlett packard-5890 series-II gas chromatograph equipped with *μ*-ECD (Ni^63^) was used for determination and chromatograms were recorded by HP (model-3396) series-II integrator and Buchi rotary evaporator (Buchi, Switzerland), Vac-Elut (Analytical Chemical International, USA), and Deep freezer (Blue Star Company, Germany) were used.

### 2.2. Chemicals and Glassware

Analytical grade metamitron of 99.2% purity was obtained from M/s PCCPL (Punjab Chemicals and Crop Protection Limited, India). All the chemicals used were of analytical reagent (AR) or HPLC grade. The glassware used was from Pyrex (USA), Schott Duran (Germany), and Borosil (India); prepacked C-18 SPE (500 mg) was purchased from Sigma-Aldrich (USA).

### 2.3. Soil

The surface soil of 0–15 cm depth was taken from N. E. Borlaug Crop Research Centre, G. B. Pant University of Agriculture and Technology, Pantnagar, and experiments were conducted in the Department of Chemistry (Division of Agricultural Chemicals), College of Basic Sciences and Humanities, G. B. Pant University of Agriculture and Technology, Pantnagar. The temperature during the experiment was 30 ± 2°C and relative humidity was 60 ± 5%. The soil used was not earlier treated with metamitron and was free from metamitron residues. The moisture of soil was maintained at 15% by weight to retain conditions as those of natural soil. The soil was also analysed for physicochemical and textural properties as per standard procedure [18].

### 2.4. Preparation of Solutions

A stock solution of metamitron (100 *μ*g mL^−1^) was prepared by dissolving 2.5 mg of metamitron in 25 mL of methanol and stored at −20°C. Working solutions of 0.05–2.0 *μ*g L^−1^ were prepared by serial dilution of the stock solution with methanol.

### 2.5. Fortification

The shade dried soil was pulverised and passed through 2 mm sieve; water was added into processed soil (50 g) for maintaining soil moisture at 15%. The soil samples were then fortified by adding a known amount (5 mL) of working standard solutions containing metamitron, so that the levels of fortification were 0.05, 0.125, 0.25, 0.50, 1.0, and 2.0 *μ*g kg^−1^.

### 2.6. Extraction

The fortified soil (2 gm) of different concentrations was taken in 50 mL conical flask. Ten mL of methanol was added into it, shaken on horizontal shaker for 30 minutes, and filtered. The process was repeated twice with fresh batch of 10 and 5 mL of methanol. The extract was pooled, added to 5 gm anhydrous sodium sulphate, and filtrate was evaporated to dryness under reduced pressure at 45 ± 1°C using Buchi rotatory flash evaporator. Residue was redissolved in 2 mL methanol and subjected to further cleanup.

### 2.7. Cleanup

The C-18 SPE minicolumn was washed with 2 mL of methanol : water (8 : 2 v/v) and washing was discarded. One mL sample extract was loaded on C-18 SPE minicolumn and washed with 1 mL water : methanol (1 : 9 v/v) and washing was discarded. The residue was finally eluted with 2 mL methanol. The aliquot was dried under the stream of nitrogen and redissolved in 1 mL HPLC grade methanol and subjected to analysis by GC-ECD.

### 2.8. Recovery Experiments

The blank soil sample and fortified soil samples (0.05–2.0 *μ*g kg^−1^) were extracted with methanol and cleaned up as described earlier. Each fortification level was extracted in triplicate and injected into GC thrice. The recovery was calculated using the ratio of area for fortified samples and the area of standard. Blank soil without metamitron was also analysed.

### 2.9. Chromatographic Conditions

The following gas chromatographic conditions were optimized to achieve proper separation for determination of metamitron at trace levels using Equity-5 microcapillary column (30 mt length, 0.25 mm ID, and 0.25 *μ*m film thickness), nitrogen at 1 mL min.^−1^ (100 KPa), and detector *μ*-ECD (Ni^63^). The oven, injector, and detector temperatures were maintained at 250°C, 275°C, and 300°C, respectively. GC was operated in splitless mode. The sample injection volume was 1 *μ*L and total run time was 12 minutes. Metamitron was identified by comparing the retention time of the peak present in the extracts of the samples with the retention time of the standard.

### 2.10. Method Validation

The method was validated by evaluating analytical curves and linearity, limit of detection (LOD), limit of quantification (LOQ), accuracy (recovery), and precision (repeatability and intermediate precision).

#### 2.10.1. Analytical Curve and Linearity

The linearity of the instrument and the method was evaluated by analytical curves with the concentration levels from the LOQ of compound, that is, 0.05 to 2.0 ng mL^−1^ with three replicate injections per concentration.

#### 2.10.2. Sensitivity, Limit of Detection, and Quantification of the Method

The sensitivity of the method was determined using ratio between the estimated standard deviation of the linear coefficient and the slope of the analytical curve. The LOD and LOQ for metamitron were determined by considering 3 and 10 times ratio of signal to noise, respectively. The obtained values were also checked experimentally.

#### 2.10.3. Precision and Accuracy

Precision in terms of repeatability was obtained by carrying out the extraction and the analysis of fortified samples. Each spiked level was extracted in three replicates and each extract was injected three times. The intermediate precision was estimated in the same way as the repeatability, but on different days. Each fortification level was extracted in triplicate and injected three times (*n* = 9).

## 3. Results and Discussion

**Figure 1:**
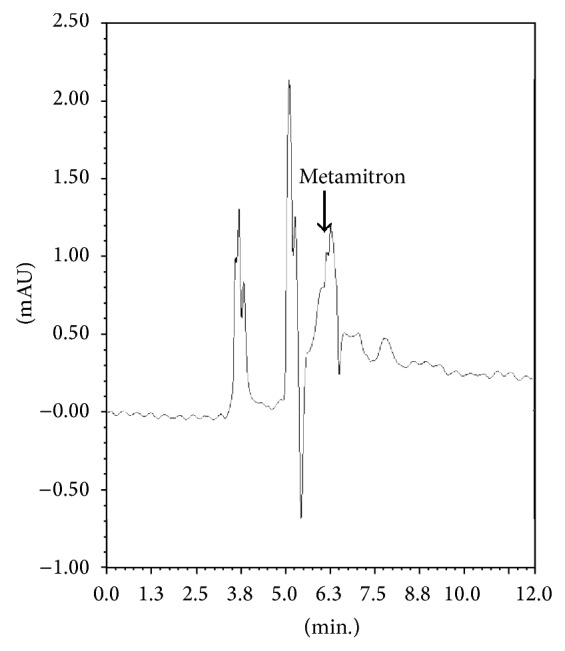
Chromatogram of metamitron extracted from soil before cleanup.

**Figure 2:**
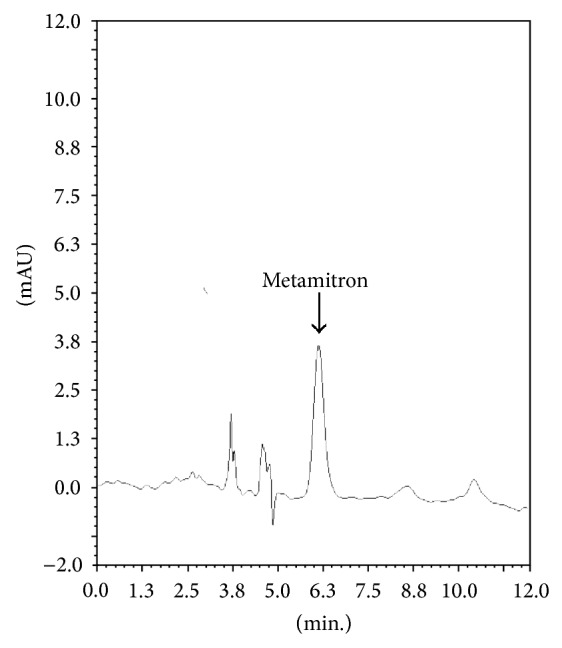
Chromatogram of metamitron extracted from soil after cleanup.

**Figure 3:**
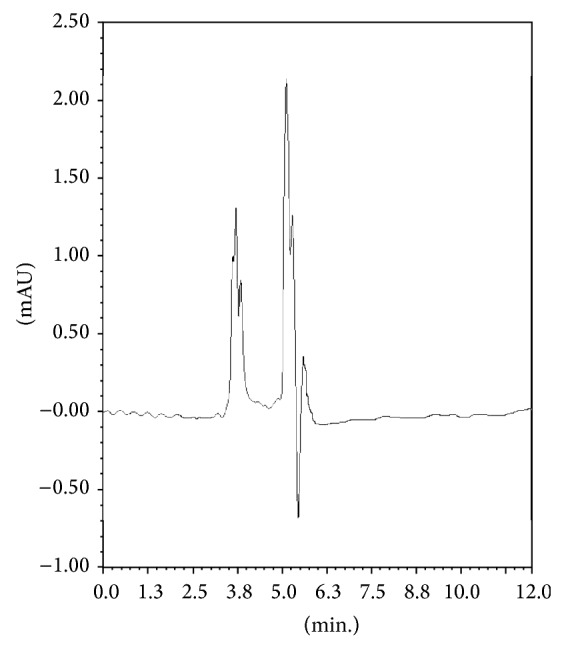
Chromatogram of blank soil.

The physicochemical properties of the soil showed that the soil was rich in organic carbon content (1.443%), loamy textured with sand : silt : clay (48.59 : 31.71 : 19.70%) ratio having neutral pH 6.94. Under the optimised GC conditions, retention time for metamitron was 6.3 minutes. In soil after cleanup no interfering peak was recorded at the retention time of metamitron showing good cleanup of the method (Figures 1 and 2). Blank soil did not contain any residue (Figure 3). The main advantage of using GC-ECD over LC methods was because it provided quicker analysis, high level of certainty to identify the object compound, and lower limit of detection.

**Figure 4:**
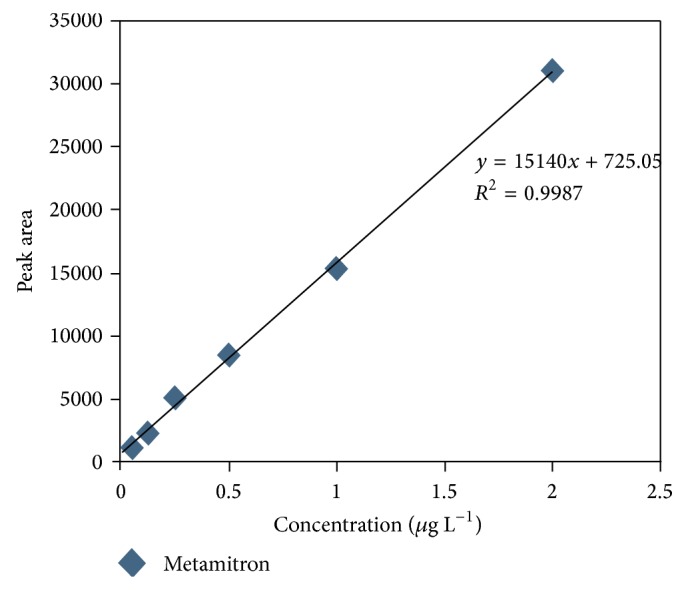
Calibration curve for metamitron.

The method was selective and showed good linearity relationship which was observed between ratio of the peak area signals and corresponding concentrations. Good determination coefficient (*R* ^2^ = 0.998) was achieved over the working concentration range of 0.05, 0.125, 0.25, 0.5, 1.0, and 2.0 ng mL^−1^ for the standard of metamitron. The analytical curve parameters of the analytical curve with the correlation coefficient are shown in Figure 4 and Table 1. When comparing the response with the baseline noise, the LOD for the metamitron was found to be 0.02 ng mL^−1^ and LOQ of method was 0.05 ng g^−1^.

The recovery under intermediate precision conditions ranged from 89.0 to 93.5% with RSD from 1.7034 to 1.9144% (Table 1). The intermediate precision ranged from 70 to 90% with RSD 1.5685%–2.1323%. All the values were in the acceptable range of recovery for trace residue analysis which is usually between 80 and 120%, RSD maximum 10%, and LOQ according to MRL (maximum residue limit) of the compound [19, 20]. These results indicate that the method is useful for the determination of metamitron at trace levels in soil samples.

Analysis of metamitron using chromatographic techniques has been done by some workers. Ghebbioni and Trevisan [9] reported 93% recovery of metamitron with LOQ 6.3 *μ*g kg^−1^ in soil by HPLC. Hongji et al. [11] analysed metamitron by HPLC with recovery of 100.19%. Metamitron estimation in soil with quantfiable limit was 0.008 *μ*g g^−1^ by HPLC which was reported by Kumar et al. [15]. Estimation in soil and sugar beet at two application rates by HPLC had been given by Janaki et al. [14]. They found metamitron persists in plant and soil up to 15 and 30 days, and average recoveries were 89 and 82%, respectively, while LOD was 0.1 *μ*g kg^−1^ in soil and root, respectively. Metamitron determination by RP-HPLC showed detection limit of 20 mg kg^−1^ and recovery at the level of 0.1 mg kg^−1^ was 93% and 87% for the soil and sugar beet, respectively, while relative standard deviations ranged from 4.0% for the soil to 3.7% in case of the plant material [12]. Kucharski et al. [16] determined metamitron residues using GC in soil and sugar beet. Residues in sugar beet root and soil samples ranged from 0.0011 to 0.0085 and 0.0085 to 0.0224 mg kg^−1^ with average recovery of 89 and 82% in soil and roots, respectively, whereas the limit of detection in both soil and roots was 0.0001 mg kg^−1^.

Our method is better than earlier reported liquid or gas chromatographic methods in terms of percent recovery (89–93.5%), RSD (1.80%), and LOQ (0.05 *μ*g kg^−1^) of the metamitron from soil matrix. The developed method here has shorter analysis time with better resolution than other studies [16] and could not be compared because the analysis time is not declared. The method is also good as it requires less time in detection of compound and uses less amount and less hazardous solvent and the method is more sensitive for detecting (0.02 ng mL^−1^) and quantification (0.05 ng g^−1^) of metamitron.

*Applicability*. The analytical method can be applied for the monitoring of pesticide residues in trace amounts by various laboratories/quality control. The method is rapid and reliable and meets the requirement for analysis of metamitron from environment matrixes such as soils.

## 4. Conclusion

The proposed method showed acceptable repeatability and provides an alternative route to determine the metamitron-like triazinone compounds from soil with a safer way with no compromise in sensitivity. Validation of the method has been shown with parameters of linearity, precision, LOD and LOQ, accuracy, and specificity. All the results of percent coefficient of variation are below 2% showing that the method was valid. The method gives more efficiency, reduced amount of chemicals, and more sensitivity in comparison to earlier reported methods. The method can be applied to determine pesticide contamination in environmental samples.

## Figures and Tables

**Table 1:** Recovery, repeatability (RSDr), intermediate precision (RSDip), and linearity of the method.

Matrix	Amount (*µ*g kg^−1^) added	Amount (*µ*g kg^−1^) recovered	Recovery (%)	RSDr%	RSDip%	Linearity
Soil	0.050	0.0445	89.0%	1.91	2.01	0.998
	0.125	0.1120	89.6%	1.90	1.83	
	0.250	0.2262	90.5%	1.89	1.75	
	0.500	0.4570	91.4%	1.78	2.13	
	0.100	0.0920	92.0%	1.74	1.69	
	1.000	0.9270	92.7%	1.71	1.57	
	2.000	1.8700	93.5%	1.70	1.98	

Average			91.24%	1.80	1.85	
